# *N*-(3-Chloro-4-fluoro­phen­yl)acetamide

**DOI:** 10.1107/S160053681201416X

**Published:** 2012-04-13

**Authors:** Hoong-Kun Fun, Wan-Sin Loh, Divya N. Shetty, B. Narayana, B. K. Sarojini

**Affiliations:** a X-ray Crystallography Unit, School of Physics, Universiti Sains Malaysia, 11800 USM, Penang, Malaysia; b Department of Studies in Chemistry, Mangalore University, Mangalagangotri 574 199, India; c Department of Chemistry, P.A. College of Engineering, Nadupadavu, Mangalore 574 153, India

## Abstract

In the title compound, C_8_H_7_ClFNO, the dihedral angle between the benzene ring and the acetamide side chain is 5.47 (6)°. An *S*(6) ring motif is formed *via* an intra­molecular C—H⋯O hydrogen bond. In the crystal, N—H⋯O hydrogen bonds link the mol­ecules into *C*(4) chains along [001].

## Related literature

For background to acetamides, see: Khan *et al.* (2010 ▶); Tahir & Shad (2011 ▶). For hydrogen-bond motifs, see: Bernstein *et al.* (1995 ▶). For a related structure, see: Rosli *et al.* (2007 ▶). For the stability of the temperature controller used in the data collection, see: Cosier & Glazer (1986 ▶).
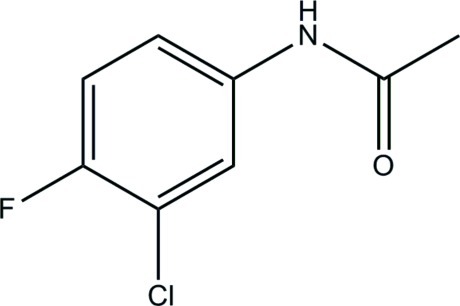


## Experimental

### 

#### Crystal data

C_8_H_7_ClFNO
*M* *_r_* = 187.60
Monoclinic, 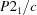

*a* = 7.6776 (4) Å
*b* = 12.7671 (7) Å
*c* = 9.8130 (4) Å
β = 124.432 (3)°
*V* = 793.35 (7) Å^3^
*Z* = 4
Mo *K*α radiation
μ = 0.44 mm^−1^
*T* = 100 K
0.33 × 0.29 × 0.15 mm

#### Data collection

Bruker SMART APEXII DUO CCD diffractometer
Absorption correction: multi-scan (*SADABS*; Bruker, 2009 ▶) *T*_min_ = 0.869, *T*_max_ = 0.937
14562 measured reflections
3971 independent reflections
3173 reflections with *I* > 2σ(*I*)
*R*_int_ = 0.035

#### Refinement

*R*[*F* ^2^ > 2σ(*F* ^2^)] = 0.047
*wR*(*F* ^2^) = 0.143
*S* = 1.09
3971 reflections
110 parameters
H-atom parameters constrained
Δρ_max_ = 1.32 e Å^−3^
Δρ_min_ = −0.50 e Å^−3^

### 

Data collection: *APEX2* (Bruker, 2009 ▶); cell refinement: *SAINT* (Bruker, 2009 ▶); data reduction: *SAINT*; program(s) used to solve structure: *SHELXTL* (Sheldrick, 2008 ▶); program(s) used to refine structure: *SHELXTL*; molecular graphics: *SHELXTL*; software used to prepare material for publication: *SHELXTL* and *PLATON* (Spek, 2009 ▶).

## Supplementary Material

Crystal structure: contains datablock(s) global, I. DOI: 10.1107/S160053681201416X/hb6705sup1.cif

Structure factors: contains datablock(s) I. DOI: 10.1107/S160053681201416X/hb6705Isup2.hkl

Supplementary material file. DOI: 10.1107/S160053681201416X/hb6705Isup3.cml

Additional supplementary materials:  crystallographic information; 3D view; checkCIF report

## Figures and Tables

**Table 1:** Hydrogen-bond geometry (Å, °)

*D*—H⋯*A*	*D*—H	H⋯*A*	*D*⋯*A*	*D*—H⋯*A*
N1—H1⋯O1^i^	0.90	2.00	2.8996 (12)	174
C1—H1*A*⋯O1	0.95	2.20	2.8222 (14)	122

Symmetry code: (i) 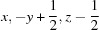.

**Fig. 1.**
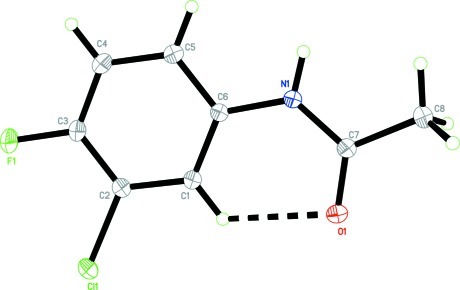
The molecular structure of the title compound, showing 50% probability displacement ellipsoids. Dashed line indicates the intramolecular hydrogen bond.

**Fig. 2.**
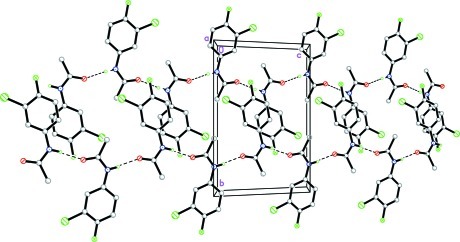
The crystal packing of the title compound, viewed along the a axis, showing the chains along the c axis. H atoms not involved in the intermolecular interactions (dashed lines) have been omitted for clarity.

## supplementary crystallographic information

### Comment

To complement earlier studies of acetamides (Khan *et al.*, 2010; Tahir & Shad, 2011), we report herein the crystal structure of the title compound.

In the title compound (Fig. 1), an *S*(6) ring motif (Bernstein *et al.*, 1995) is formed *via* intramolecular C1—H1A···O1 hydrogen bond (Table 1). Bond lengths and angles are within the normal ranges and are comparable with the related structure (Rosli *et al.*, 2007).

In the crystal (Fig. 2), N1—H1···O1 hydrogen bonds (Table 1) link the molecules to form chains along the *c* axis.

### Experimental

3-Chloro-4-fluoro aniline (0.145 g, 1 mmol) was dissolved in acetic acid (20 mL) and refluxed for 4 h. The solution was then cooled and poured into 100 ml of ice-cold water with stirring. The precipitate obtained was filtered, washed with water and dried. Orange blocks were grown from DMF solution by the slow evaporation method. *M. P.*: 384 K.

### Refinement

N-bound H atoms were located from the difference Fourier map and were refined with a riding model with *U*_iso_(H) = 1.2 *U*_eq_(N) [N–H = 0.9003 Å]. The remaining H atoms were positioned geometrically and refined with a riding model with *U*_iso_(H) = 1.2 or 1.5 *U*_eq_(C) [C–H = 0.95 or 0.98 Å]. A rotating group model was applied to the methyl groups. In the final refinement, five outliners were omitted, -3 8 2, -1 0 1, -3 8 1, 1 0 0 and -1 0 4. In the final difference Fourier map, the highest peak and the deepest hole are 0.83 and 0.71Å from atom Cl1.

### Crystal data

**Table:**

C_8_H_7_ClFNO	*F*(000) = 384
*M**_r_* = 187.60	*D*_x_ = 1.571 Mg m^−^^3^
Monoclinic, *P*2_1_/*c*	Mo *K*α radiation, λ = 0.71073 Å
Hall symbol: -P 2ybc	Cell parameters from 5541 reflections
*a* = 7.6776 (4) Å	θ = 3.0–36.8°
*b* = 12.7671 (7) Å	µ = 0.44 mm^−^^1^
*c* = 9.8130 (4) Å	*T* = 100 K
β = 124.432 (3)°	Block, orange
*V* = 793.35 (7) Å^3^	0.33 × 0.29 × 0.15 mm
*Z* = 4	

### Data collection

**Table:**

Bruker SMART APEXII DUO CCD diffractometer	3971 independent reflections
Radiation source: fine-focus sealed tube	3173 reflections with *I* > 2σ(*I*)
Graphite monochromator	*R*_int_ = 0.035
φ and ω scans	θ_max_ = 36.9°, θ_min_ = 3.2°
Absorption correction: multi-scan (*SADABS*; Bruker, 2009)	*h* = −12→12
*T*_min_ = 0.869, *T*_max_ = 0.937	*k* = −21→17
14562 measured reflections	*l* = −14→16

### Refinement

**Table:**

Refinement on *F*^2^	Primary atom site location: structure-invariant direct methods
Least-squares matrix: full	Secondary atom site location: difference Fourier map
*R*[*F*^2^ > 2σ(*F*^2^)] = 0.047	Hydrogen site location: inferred from neighbouring sites
*wR*(*F*^2^) = 0.143	H-atom parameters constrained
*S* = 1.09	*w* = 1/[σ^2^(*F*_o_^2^) + (0.0844*P*)^2^ + 0.1402*P*] where *P* = (*F*_o_^2^ + 2*F*_c_^2^)/3
3971 reflections	(Δ/σ)_max_ < 0.001
110 parameters	Δρ_max_ = 1.32 e Å^−^^3^
0 restraints	Δρ_min_ = −0.50 e Å^−^^3^

### Special details

**Table:**

Experimental. The crystal was placed in the cold stream of an Oxford Cryosystems Cobra open-flow nitrogen cryostat (Cosier & Glazer, 1986) operating at 100.0 (1) K.
Geometry. All e.s.d.'s (except the e.s.d. in the dihedral angle between two l.s. planes) are estimated using the full covariance matrix. The cell e.s.d.'s are taken into account individually in the estimation of e.s.d.'s in distances, angles and torsion angles; correlations between e.s.d.'s in cell parameters are only used when they are defined by crystal symmetry. An approximate (isotropic) treatment of cell e.s.d.'s is used for estimating e.s.d.'s involving l.s. planes.
Refinement. Refinement of *F*^2^ against ALL reflections. The weighted *R*-factor *wR* and goodness of fit *S* are based on *F*^2^, conventional *R*-factors *R* are based on *F*, with *F* set to zero for negative *F*^2^. The threshold expression of *F*^2^ > σ(*F*^2^) is used only for calculating *R*-factors(gt) *etc*. and is not relevant to the choice of reflections for refinement. *R*-factors based on *F*^2^ are statistically about twice as large as those based on *F*, and *R*- factors based on ALL data will be even larger.

### Fractional atomic coordinates and isotropic or equivalent isotropic displacement parameters (Å^2^)

**Table:**

	*x*	*y*	*z*	*U*_iso_*/*U*_eq_	
Cl1	0.32215 (5)	−0.12199 (2)	0.38846 (3)	0.02251 (9)	
F1	0.31780 (12)	−0.24025 (5)	0.13350 (9)	0.02207 (16)	
O1	0.18905 (15)	0.24972 (7)	0.21236 (10)	0.02070 (17)	
N1	0.20506 (15)	0.18521 (7)	0.00234 (11)	0.01588 (16)	
H1	0.1979	0.2007	−0.0902	0.019*	
C1	0.27231 (17)	0.03769 (8)	0.18824 (12)	0.01625 (18)	
H1A	0.2766	0.0831	0.2668	0.019*	
C2	0.29704 (17)	−0.06971 (8)	0.21576 (13)	0.01634 (18)	
C3	0.29615 (17)	−0.13592 (8)	0.10379 (13)	0.01655 (18)	
C4	0.27098 (18)	−0.09670 (9)	−0.03746 (13)	0.01834 (19)	
H4A	0.2733	−0.1423	−0.1129	0.022*	
C5	0.24229 (17)	0.01024 (9)	−0.06795 (13)	0.01731 (19)	
H5A	0.2233	0.0377	−0.1655	0.021*	
C6	0.24111 (16)	0.07801 (8)	0.04398 (12)	0.01421 (17)	
C7	0.17661 (17)	0.26277 (8)	0.08257 (13)	0.01566 (18)	
C8	0.1266 (2)	0.36891 (9)	0.00122 (14)	0.0196 (2)	
H8A	0.2467	0.4160	0.0687	0.029*	
H8B	0.0013	0.3977	−0.0091	0.029*	
H8C	0.0992	0.3619	−0.1088	0.029*	

### Atomic displacement parameters (Å^2^)

**Table:**

	*U*^11^	*U*^22^	*U*^33^	*U*^12^	*U*^13^	*U*^23^
Cl1	0.03690 (18)	0.01515 (13)	0.02113 (14)	0.00050 (9)	0.01980 (13)	0.00337 (8)
F1	0.0318 (4)	0.0111 (3)	0.0247 (3)	0.0014 (2)	0.0167 (3)	0.0004 (2)
O1	0.0340 (4)	0.0164 (4)	0.0183 (3)	0.0011 (3)	0.0188 (3)	−0.0001 (3)
N1	0.0246 (4)	0.0120 (3)	0.0155 (3)	−0.0003 (3)	0.0140 (3)	0.0001 (3)
C1	0.0230 (5)	0.0126 (4)	0.0160 (4)	−0.0006 (3)	0.0128 (4)	−0.0006 (3)
C2	0.0214 (4)	0.0136 (4)	0.0162 (4)	−0.0009 (3)	0.0120 (3)	0.0003 (3)
C3	0.0204 (4)	0.0116 (4)	0.0183 (4)	0.0000 (3)	0.0114 (4)	−0.0005 (3)
C4	0.0243 (5)	0.0152 (4)	0.0180 (4)	0.0002 (3)	0.0134 (4)	−0.0029 (3)
C5	0.0238 (5)	0.0153 (4)	0.0165 (4)	−0.0002 (3)	0.0136 (4)	−0.0011 (3)
C6	0.0183 (4)	0.0122 (4)	0.0148 (4)	−0.0007 (3)	0.0109 (3)	−0.0003 (3)
C7	0.0201 (4)	0.0134 (4)	0.0159 (4)	−0.0007 (3)	0.0116 (3)	−0.0003 (3)
C8	0.0281 (5)	0.0136 (4)	0.0211 (5)	0.0011 (3)	0.0163 (4)	0.0019 (3)

### Geometric parameters (Å, º)

**Table:**

Cl1—C2	1.7278 (11)	C3—C4	1.3807 (15)
F1—C3	1.3535 (12)	C4—C5	1.3884 (15)
O1—C7	1.2335 (13)	C4—H4A	0.9500
N1—C7	1.3573 (14)	C5—C6	1.4023 (14)
N1—C6	1.4102 (14)	C5—H5A	0.9500
N1—H1	0.9003	C7—C8	1.5081 (15)
C1—C2	1.3896 (15)	C8—H8A	0.9800
C1—C6	1.3938 (14)	C8—H8B	0.9800
C1—H1A	0.9500	C8—H8C	0.9800
C2—C3	1.3832 (15)		
			
C7—N1—C6	127.56 (9)	C4—C5—C6	120.56 (10)
C7—N1—H1	119.1	C4—C5—H5A	119.7
C6—N1—H1	113.3	C6—C5—H5A	119.7
C2—C1—C6	119.21 (9)	C1—C6—C5	119.61 (10)
C2—C1—H1A	120.4	C1—C6—N1	123.06 (9)
C6—C1—H1A	120.4	C5—C6—N1	117.32 (9)
C3—C2—C1	120.66 (10)	O1—C7—N1	123.81 (10)
C3—C2—Cl1	119.39 (8)	O1—C7—C8	121.05 (10)
C1—C2—Cl1	119.93 (8)	N1—C7—C8	115.14 (9)
F1—C3—C4	120.24 (9)	C7—C8—H8A	109.5
F1—C3—C2	119.04 (10)	C7—C8—H8B	109.5
C4—C3—C2	120.71 (10)	H8A—C8—H8B	109.5
C3—C4—C5	119.22 (10)	C7—C8—H8C	109.5
C3—C4—H4A	120.4	H8A—C8—H8C	109.5
C5—C4—H4A	120.4	H8B—C8—H8C	109.5
			
C6—C1—C2—C3	1.61 (16)	C2—C1—C6—C5	−2.14 (16)
C6—C1—C2—Cl1	−176.64 (8)	C2—C1—C6—N1	177.04 (10)
C1—C2—C3—F1	−179.12 (10)	C4—C5—C6—C1	1.00 (16)
Cl1—C2—C3—F1	−0.85 (14)	C4—C5—C6—N1	−178.22 (10)
C1—C2—C3—C4	0.09 (16)	C7—N1—C6—C1	−6.20 (17)
Cl1—C2—C3—C4	178.35 (9)	C7—N1—C6—C5	172.99 (10)
F1—C3—C4—C5	177.95 (10)	C6—N1—C7—O1	3.71 (18)
C2—C3—C4—C5	−1.24 (17)	C6—N1—C7—C8	−176.05 (10)
C3—C4—C5—C6	0.69 (17)		

### Hydrogen-bond geometry (Å, º)

**Table:**

*D*—H···*A*	*D*—H	H···*A*	*D*···*A*	*D*—H···*A*
N1—H1···O1^i^	0.90	2.00	2.8996 (12)	174
C1—H1*A*···O1	0.95	2.20	2.8222 (14)	122

Symmetry code: (i) *x*, −*y*+1/2, *z*−1/2.
